# Treatment of atypical gouty arthritis of the hip using total hip arthroplasty: A case report

**DOI:** 10.1097/MD.0000000000023027

**Published:** 2020-10-30

**Authors:** Yuyang Huang, Jiongfeng Huang, Cheng Luo, Li’an Chen, Bingsheng Huang

**Affiliations:** Department of Joint Surgery, Guangzhou Panyu Central Hospital, Guangzhou, China.

**Keywords:** avascular necrosis of the femoral head, hip gout arthritis, pathology, total hip arthroplasty

## Abstract

**Rationale:**

Gout and gouty arthritis typically involve peripheral and monoarticular joints, especially the first metatarsophalangeal joint and knees. Hip involvement in patients with gout is rare, and its diagnosis is very difficult, especially in the late stages of the disease. Total hip arthroplasty could be a surgical treatment for atypical gouty arthritis of the hip; however, few cases have been reported.

**Patient concerns:**

We reported an uncommon case of a 74-year-old man without typical symptoms of hip gout arthritis whom was misdiagnosed as having avascular necrosis of the femoral head.

**Diagnoses:**

Clinical examination and imaging revealed bilateral avascular necrosis of the femoral head. However, the final pathology report revealed left hip gout arthritis.

**Interventions:**

The patient underwent left total hip arthroplasty and was followed up for 3years.

**Outcomes:**

The outcome was favorable. The function of the left hip was almost normal.

**Lessons:**

Our case indicated the difficulty of the diagnosis of hip gout arthritis. Due to the lower rates of hip gout arthritis and lack of typical clinical examination, it is easy to misdiagnose. Furthermore, surgical treatment for the late stage of hip gout arthritis has not previously been reported. In our case, total hip arthroplasty proved to be a good option.

## 1 Introduction

Gout is a common rheumatologic disease characterized by painful flares during the acute arthritis and asymptomatic chronic phase. Gout is an inflammatory arthritis characterized by recurrent attacks of red, tender, hot, and swollen joints.^[1]^ Gouty arthritis typically involves peripheral and monoarticular joints, especially the first metatarsophalangeal joint and knees.^[1,2]^ Anti-inflammatories, reduction, and excretion of uric acid are the standard medical therapies for gout.^[3]^ For the most commonly affected joints in gout, including the first metatarsophalangeal joint, ankle, and knee, tophus excision^[4]^ and arthroscopic operations are the most common surgical treatments. By contrast, gout of the hip is rare and its diagnosis is very difficult.^[5]^ There are few reports of hip gout arthritis that consist only of ultrasound and clinical features, and none of the reported cases involve severe joint dysfunction requiring total hip arthroplasty.^[5,6]^ We here reported an unusual case of hip gout arthritis without typical clinical symptoms, but with severe loss of joint function. In the present rare case, we aimed to provide a reference for the diagnosis and surgical treatment of hip gout arthritis.

## 2 Case presentation

**Figure 1:**
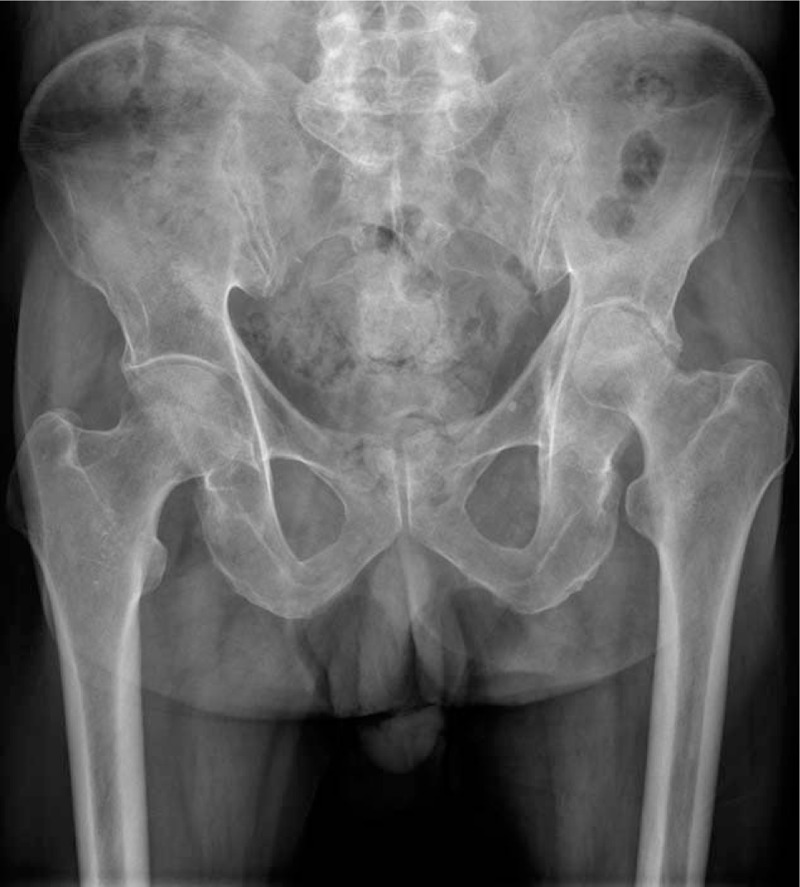
The preoperative X-ray of the pelvis. Preoperative X-ray of the pelvis. The left femoral head was dislocated and collapsed.

**Figure 2:**
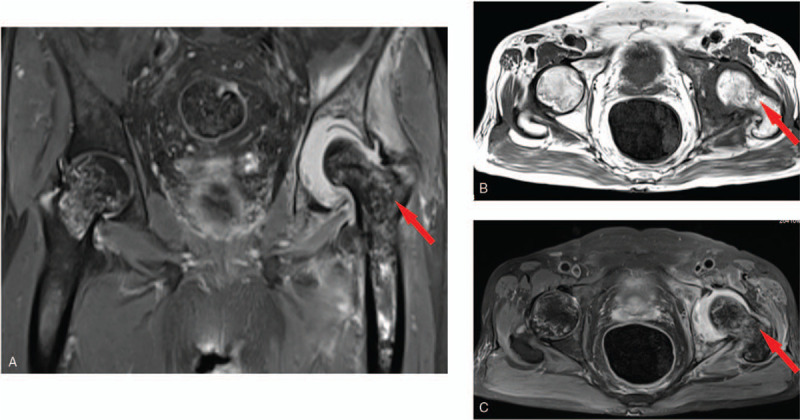
Preoperative magnetic resonance imaging of both hip joints. Preoperative magnetic resonance imaging of both hip joints. A, coronal plane MRI of hip joints by showed that there were abnormal signal in both femoral head and left femoral medullary cavity, which was consistent with changes of avascular necrosis of the femoral head. The arrow indicates the damaged left acetabular notch, and large defects with joint effusion were found. B, C, transverse-ligament MRI images also show the changes of avascular necrosis of both femoral head with obvious effusion of the left hip joint.

A 74-year-old man presented with left hip and right ankle pain for a month. He self-medicated with non-steroidal anti-inflammatory drugs, which improved his pain. Two weeks later, the symptoms worsened, with redness and swelling of the left ankle and both knees, and limited left hip function, while the other joints remained normal. He had been an alcoholic for 20 years and denied smoking. On his medical history, he had spent 20 years without formal treatment. On physical examination, the patient had a significant limp and limited mobility at the left hip joint. The left limb was shorter than the other one by about 1.5 cm. Tenderness was observed 2 cm below the midpoint of the left inguinal ligament. Laboratory data revealed a uric acid(UA) level of 534 μmol/L, white blood cell count of 8.58 × 10^9^/L, erythrocyte sedimentation rate(ESR) of 124 mm/h, and C-reactive protein(CRP) level of 2.4 mg/L. There were no detectable tuberculosis antibodies, infectious indicators, or tumor markers. The radiograph of the pelvis revealed that the left femoral head had undergone cystic degeneration and deformation (Fig. 1). Magnetic resonance imaging (MRI) revealed high signal intensity around the left acetabulum and high signal intensity of a patchy area in the left femoral canal. The left femoral head had an irregular shape and collapse (Fig. 2A–C). The patient's provisional diagnosis was bilateral avascular necrosis of the femoral head and gouty arthritis of both knees and ankles.

After controlling the level of uric acid, we performed left hip replacement. The posterolateral approach was used for the left hip. When we opened the hip capsule, there were full of ash-colored liquid containing granules of sesame-like size. The bones in the inner top and inferior part of the acetabular notch were soft and rotted, but without caseous necrosis or fish meat-like tumor tissue. We sent a number of damaged tissues for pathological analysis. Then, we removed the diseased tissue of the acetabulum until we reached bone with normal hardness. The next step was bone grafting to the defect area, osteotomy, enlarging the femoral medullary cavity, and implanting the prosthesis for hip replacement as per routine. The prosthesis was an ultra-high molecular weight polyethylene acetabular cup and a biological femoral prosthesis. Postoperatively, we administered benzbromarone (50 mg/day) and allopurinol (100 mg/day) to control the level of uric acid. Cefuroxime (3 g/day) was used for 48 hours to prevent infection. One day after the surgery, the patient was able to walk from the bed. The wound healed well, and stitches were removed 14 days after surgery.

**Figure 3:**
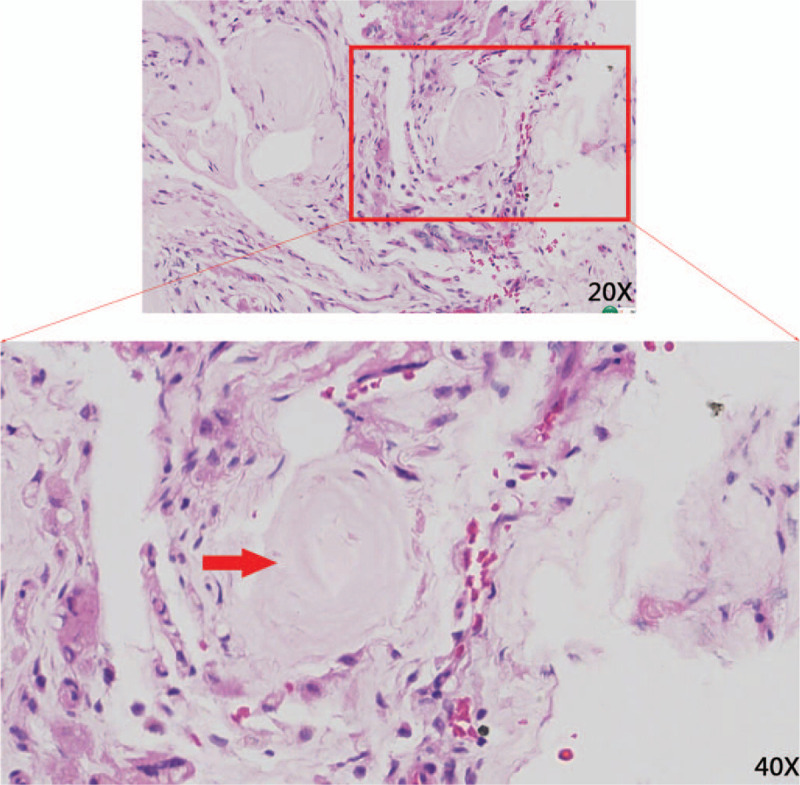
Histopathological evaluation of a section of the left hip necrotic tissue. Histopathological evaluation of a section of the left hip necrotic tissue. A, the red box (magnification: 20×) and the red arrow(magnification: 40×)showed pink stained crystals with no cellular structure surrounded by multinucleated giant cells, chronically inflamed cells, and fibrillations being suggestive for gouty tophus.

**Figure 4:**
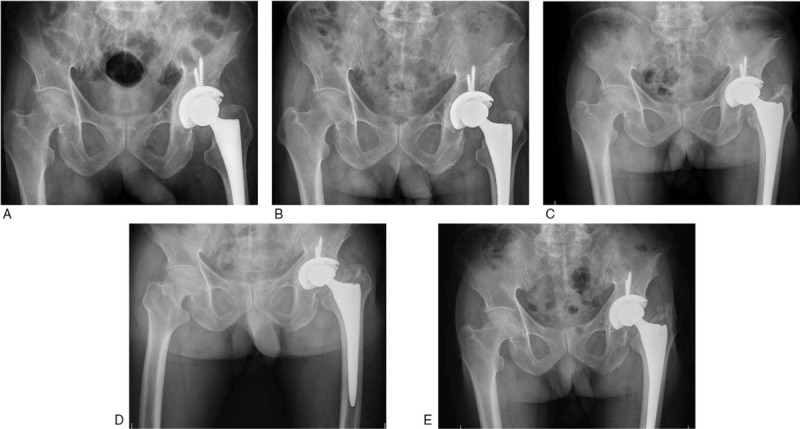
Postoperative follow-up through CR of plain pelvic films. Postoperative follow-up through CR of plain pelvic films. A–E showing pelvic radiographs at 1, 3, 12, 20, and 32 months after surgery, respectively. The left hip prosthesis with good healing of the bone graft without any complication.

The pathology report revealed that dead bone and urate crystals were observed in the necrotic tissue, confirming the diagnosis of gouty arthritis of the left hip (Fig. 3). The patient was followed up for three years, with no implant infection or recurrence of arthritis symptoms in the left hip. Follow-up computed radiography (CR) of the pelvis revealed that the position of the left hip prosthesis was favorable and the surrounding bone was excellent (Fig. 4A–E). The function of the left hip was almost normal.

## 3 Discussion

Gouty arthritis is a common rheumatoid arthritis caused by deposition of monosodium urate (MSU).^[5]^ It often appears in middle-aged men, mainly occurring asymmetrically in the peripheral joints, especially in the first metatarsophalangeal joint, knee, and ankle.^[2,5,7]^ However, involvement of the hip is atypical and rare.^[5,8]^

To our knowledge, this is one of the few cases in which gout occurs in the hip joint and leads to joint damage. In our case, the patient had acute polyarthritis during the course of the disease. The ankle and knees were swollen and painful, which are typical symptoms of gouty arthritis. However, the left hip only showed bearable pain and limited function, without redness and swelling of the joint. Laboratory data revealed a high levels of ESR and serum acid uric acid, while there were no obvious abnormal signs of tuberculosis antibody, infectious indicators, or tumor markers. Radiographic imaging techniques, including X-ray and MRI suggested bilateral avascular necrosis of femoral head, as well as acetabular bone deficiency and joint fluid in the left hip. Moreover, the patient had a history of alcohol abuse, which was one of the causes of avascular necrosis of the femoral head. As a result, the diagnosis of infectious disease and tumor had been eliminated, and the preoperative diagnosis was bilateral avascular necrosis of the femoral head. After controlling the level of uric acid, we performed preoperative preparation for total hip arthroplasty (THA). During the operation, we found that the left hip joint capsule was full of gray lime-like secretions, and the pathology report indicated gouty arthritis with osteonecrosis.

The present case revealed difficulties in the diagnosis of hip gout arthritis. First, on the basis of cases reported worldwide, we suggest that the hip joint affected by attacks of gout may have no typical symptoms of acute joint swelling, severe pain, or increase in skin temperature. Second, under normal physiological conditions and temperature, the concentration of uric acid in blood could be maintained at a normal level.^[9]^ However, at lower pH or temperature, the solubility of monosodium urate was remarkably lower. This could explain why gouty arthritis more often affects peripheral weight-bearing joints (e.g., the first metatarsophalangeal joint, knee, and ankle).^[7]^ By contrast, the hip joint is closer to the heart, and it had superior perfusion and body temperature, resulting in a lower incidence of gouty arthritis. Thus, we could easily ignore the possibility of gouty arthritis due to our traditional thinking pattern. Tophus pressure, chemokines, pro-inflammatory cytokines, and matrix-degrading cells from tophi could lead to osteoarticular changes. Radiographic examination, including plain radiography, computed tomography (CT), and MRI might show the bony and joint lesions characteristic of gout.^[10]^ However, the early stage of gout had no characteristic of imageology appearance. In the middle and late stages of gout, deposition of tophi is often associated with osteoarthritis.^[7]^ Consequently, bone and joint lesions of osteoarthritis, such as hyperosteogeny, osteophyte formation, and cystic change, might affect the diagnosis of MSU deposition. However, a previous study suggested that hip gouty arthritis could show the typical double-contour sign in ultrasound.^[11]^ Another study indicated that MSU crystals could be observed as green on dual-energy computed tomography (DECT). These findings might be helpful for the diagnosis of hip gout arthritis to a certain extent.

Because there are few reports on the surgical treatment of the late stage of hip gout arthritis domestically, we shared some surgical experience from our case. First, we found that the late stage of gouty arthritis of the hip may be accompanied by a large bone defect in the acetabulum. Thus, bone grafting should be considered during surgery. Second, THA could be a surgical treatment for gouty arthritis of the hip with severe joint function defects, while the level of uric acid was controlled. Finally, postsurgical treatment including hip function training and wound care could follow routine protocol after THA.

In conclusion, it is difficult for clinicians to make a precise diagnosis due to a lack of typical clinical symptoms, signs, and accessory examinations. As a result, for a number of patients with hyperuricemia with or without hip symptoms, the possibility of gouty arthritis of the hip should be considered. Except for routine blood tests and imaging examinations, we suggest that hip ultrasound examination and joint fluid analysis of MSU crystals should be implemented as regular examinations. In terms of treatment, we suggest that control of uric acid levels is particularly important in the early stage of gouty arthritis. When the patient enters into the late stage of gouty arthritis of the hip with severe joint dysfunction, THA might be an excellent option.

## Author contributions

**Conceptualization:** Bingsheng Huang.

**Data curation:** Li’an Chen.

**Investigation:** Cheng Luo.

**Writing – original draft:** Yuyang Huang.

**Writing – review & editing:** Jiongfeng Huang, Bingsheng Huang.
